# A 4D view on mRNA

**DOI:** 10.18632/oncotarget.5121

**Published:** 2015-08-10

**Authors:** Carlas Smith, Li-Chun Tu, Grunwald David

**Affiliations:** RNA Therapeutics Institute and Department of Biochemistry and Molecular Pharmacology, University of Massachusetts Medical School, Worcester, MA, USA

**Keywords:** 3D microscopy, biophysics, mRNA trafficking, single molecule imaging

Imaging single molecules in live cells in 4^+^D (space, time and colors) is crucial for studying various biological processes, especially for observing the behavior of RNA molecules within the nuclear landscape [1]. RNA molecules are known to serve a multitude of tasks such as being templates for protein translation or to act as enzymes for regulating countless reactions in the nucleus [1]. Studying RNA kinetics in living cells can provide new information on RNA function or even human diseases, for instance caused by viruses such as the human immunodeficiency virus (HIV) [2]. A challenge to imaging nuclear RNA function is that the nucleus as a whole undergoes major reformation during the cell cycle [1] but the time required to step through the sample limits the capability to image large numbers of rapidly moving particles in a 3D space.

**Figure 1:**
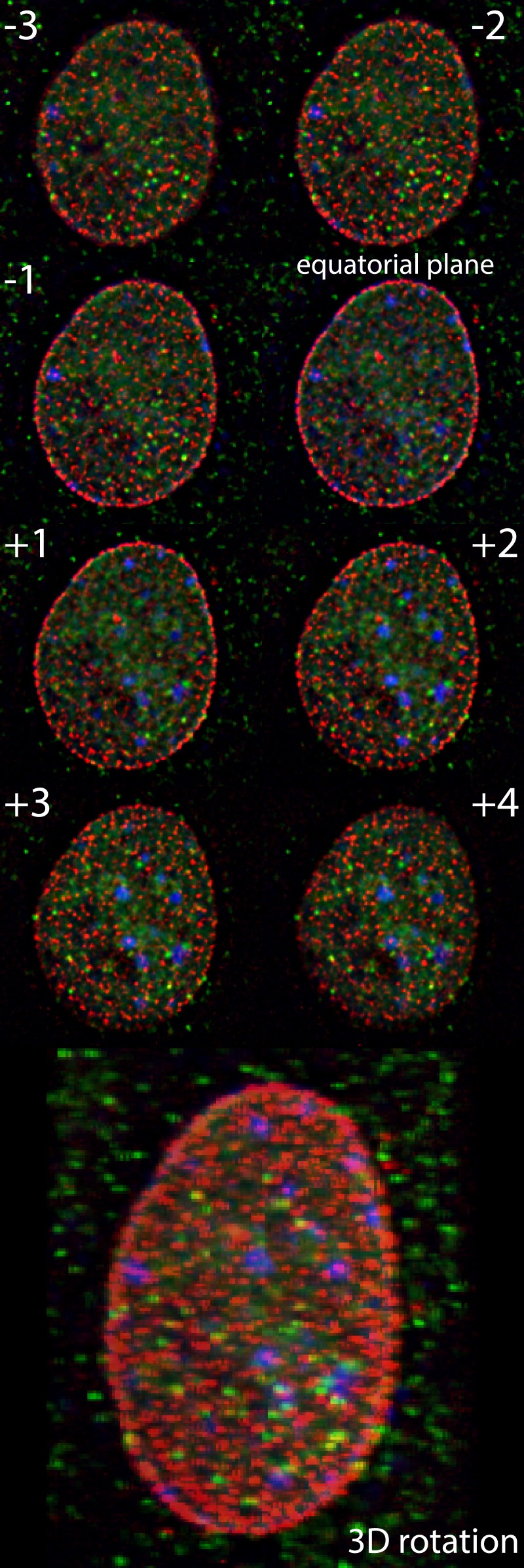
3D SMRT reconstructed 3D image after deconvolution showing nuclear pores (red), β-actin mRNA (green) and DNA (blue) Image z-planes as indicated.

Projecting information from a 3D volume into a single image plane, for example, using astigmatism, double helix spiral phase microscopy or similar Fourier-space engineered approaches [3], can circumvent sequential z-stack imaging. However, these techniques also introduce constrains on sample properties beyond those already related to imaging of single RNAs, as they rely on high signal to noise ratio and cannot function properly when single molecules are clustered. Biplane or multifocus microscopy circumvents these constraints by imaging at a greater depth of focus using multiple planes at different focal positions [3]. Here we applied multifocus microscopy (MFM) to simultaneously capture the entire nucleus of a live cell by using 3D single-molecule real-time (3D-SMRT) images with a frame rate of 10 volumes per second (Figure 1) [6].

In order to reliably identify and track individual RNA molecules, the image reconstruction from the acquired 3D snapshots is critical and non-trivial [4]. We solve this issue by using a special live cell DNA stain that is excited with low intensity in the blue spectral range and emits strongly over a broad range from 400 to 800 nm. We developed image registration tools to align the instantaneously captured image planes from the 3D volume and to accurately identify the location of each of the image planes in the 3^rd^ dimension (axial - *z*) [4]. The newly developed registration method not only corrects the chromatic aberrations but also improves the sample-induced aberrations with a remaining error of 80 nm.

The 3D-SMRT is a powerful tool to answer fundamental questions that cannot be addressed by conventional microscopy, such as visualizing various RNA regulatory processes in living cells. In our work, we imaged the nuclei of live mouse fibroblasts expressing fluorescently labeled nuclear pores and β*-actin* mRNA [4]. We found that β*-actin* mRNAs are able to access every part of the nucleus freely and that the transcripts were not excluded from heterochromatin-rich regions. Furthermore, most mRNAs were located within 0.5 μm of a nuclear pore, caused by the nucleus' disc-like shape which can only be observed in 3D images.

Going forward, we use 3D-SMRT to study how dynamic movements of nuclear products like RNA relate to the nuclear structure and function. One prominent reporter system is the HIV-1 viral genome. HIV-1 RNAs have a minimal set of proteins that are crucial to its function [2], but it hitchhikes many of the host pathways in order to export from the nucleus and form virions. Comparing the behavior of HIV genomes in living cells to that of β-actin housekeeping mRNA [5] by analyzing diffusion speed, potential interaction sites, interaction partners, interaction durations, dimerization, and modes of exiting the cell nucleus, provides important information on HIV export and latency [2, 6]. Another exciting approach lies in new gene labeling methods based on the CRISPR system to directly target the structure of chromosomes in the nucleus of living human cells [7]. The ability to observe gene expression on different chromosomes in live cells in real-time will undoubtedly add a new perspective to how we perceive RNA regulatory reactions like RNA splicing or transport, and the way the biological function of DNA and RNA regulators are executed. 4^+^D microscopy will enable us to define the rules that govern the kinetics, locations, and interactions of proteins and nuclear acids that might be exploited for the treatment of human disease.
